# Machine Learning Model Application and Comparison in Actuated Traffic Signal Forecasting

**DOI:** 10.3390/s23156912

**Published:** 2023-08-03

**Authors:** Feng Xie, Sebastian Naumann, Olaf Czogalla, Hartmut Zadek

**Affiliations:** Institut für Automation und Kommunikation e.V., 39106 Magdeburg, Germany

**Keywords:** intelligent transport, time-series, traffic signals prediction, machine learning, V2I communication, IoT

## Abstract

Traffic signal forecasting plays a significant role in intelligent traffic systems since it can predict upcoming traffic signal without using traditional radio-based direct communication with infrastructures, which causes high risk in the communication security. Previously, mathematical and statistical approach has been adopted to predict fixed time traffic signals, but it is no longer suitable for modern traffic-actuated control systems, where signals are dependent on the dynamic requests from traffic flows. And as a large amount of data is available, machine learning methods attract more and more attention. This paper views signal forecasting as a time-series problem. Firstly, a large amount of real data is collected by detectors implemented at an intersection in Hanover via IoT communication among infrastructures. Then, Baseline Model, Dense Model, Linear Model, Convolutional Neural Network, and Long Short-Term Memory (LSTM) machine learning models are trained by one-day data and the results are compared. At last, LSTM is selected for a further training with one-month data producing a test accuracy over 95%, and the median of deviation is only 2 s. Moreover, LSTM is further evaluated as a binary classifier, generating a classification accuracy over 92% and AUC close to 1.

## 1. Introduction

**Figure 1:**
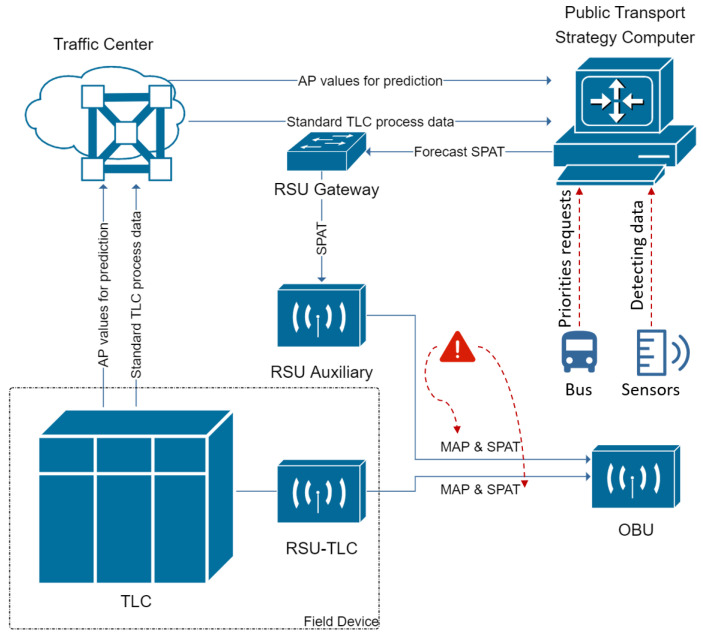
V2I communication in ITS based on IoT.

Due to a rapid development of modern traffic, there is an explosion of traffic flows. As a result, more and more air pollution and wasted energy are caused by stop-and-go driving behaviours at intersections. To mitigate such problems, some smart applications have been developed, e.g., Green Light Optimal Speed Advisory (see [1]) for vehicles to avoid unnecessary stops at signalized intersections. The precondition for all these smart applications is that the signals must be known in advance. Normally, the information can be obtained by Signal Phase and Timing messages broadcast by road side units in modern Cooperative Intelligent Transport Systems. As shown in Figure 1, it is a basic communication structure for V2I (Vehicle to Infrastructure) based on IoT (Internet of Things). The field device is constructed by a TLC (Traffic Light Controller) connected with a RSU (Road Side Unit), which first broadcasts current MAP (map as intersection geometry) and SPAT (Signal Phase and Timing) messages. Then, the traffic center delivers some application data from TLC to the public transport strategy computer, which finally generates future calculated MAP and SPAT messages. Therefore, both the current and future SPAT messages can be received by an OBU (On-Board Unit) implemented in a vehicle for further processing. However, more traffic flows are competing to request the signal messages, such as autonomous vehicles and public transport. The future traffic signals can be affected by sensors detecting vehicles in line. On the one hand, in such case, the priority of public transport cannot be guaranteed definitely. On the other hand, it increases the risk of such a radio-based communication and the cost of large amounts of communication modules implemented in intelligent transport systems. Therefore, methods to predict future traffic signals to avoid a heavy direct communication with infrastructures are being explored.

Previously, the main method to forecast upcoming traffic signal was the mathematical and statistical approach. Wang et al. [2] used Kalman Filter to predict traffic state. Menig et al. [3] adopted Markow chains to calculate the probabilities of occurrence of several signal states. However, these approaches can only produce unsatisfactory accuracy and transportability for actuated traffic systems, in which traffic signal changes are dependent on the requests from different traffic flows. Later, due to the explosion of the large data pool collected by different detectors, machine learning models attracted more attention [4]. Weisheit and Hoyer [5] applied Support Vector Machines to predict future possible traffic states, where the states were divided into different possible groups for classification. Heckmann et al. [6] further defined stages to group-related signal states that can forecast three states in advance. The authors viewed signal prediction as a regression problem, and compared the performance of different combinations of Extreme-Gradient-Boosting and Bayesian Networks (see [7]). However, these works have to assume that the traffic cycle time is fixed, which is not applicable for actuated traffic signals. Another research perspective is to view signal prediction as a time-series forecasting problem [8,9]. Khosravi et al. [10] used machine learning to predict time-series wind speed data of a wind farm in Brazil. The researchers compared Adaptive Neuro-Fuzzy Inference System and hybrid models, Multilayer Feed-Forward Neural Network, Support Vector Regression, Fuzzy Inference System, and Group Method of Data Handling type neural network, which provided a possibility to deal with traffic signals as time-series data. Genser et al. [11] made efforts to standardize Signal Phase and Timing messages to forecast the residue time of each phase. They applied a Random Survival Forest model to forecast time to green compared with the baseline models of Auto-Regressive Integrated Moving Average and Linear Regression. They mentioned the high potential of the Long Short-Term Memory (LSTM) model dealing with such time-series problem. Zhou et al. [12] proposed the Informer to solve the problem of long sequence time-series forecasting. It is a modified Transformer that increases the prediction capacity. It was successfully applied to predict electricity consumption for a long period. Tang et al. [13] rethought one-dimensional convolutional neural networks (1D-CNNs) from the omni-scale for time-series classification tasks and provided a stronger baseline. Therefore, this research explores some machine learning methods to predict future traffic signals as time-series data. The LSTM, Baseline Model, Linear Model, Dense Model, and Convolutional Neural Network are applied and compared for traffic signal forecasting in this work.

The rest of this paper is organized as follows. Section 2 introduces the ways in which the collected data are processed, as well as the basic structure of the researched machine learning models. Section 3 describes forecasting results and makes further analysis on the test accuracy and basic metrics for different time horizons. Section 4 discusses the results and provides future research direction.

## 2. Materials and Methods

### 2.1. Data Preparation

**Figure 2:**
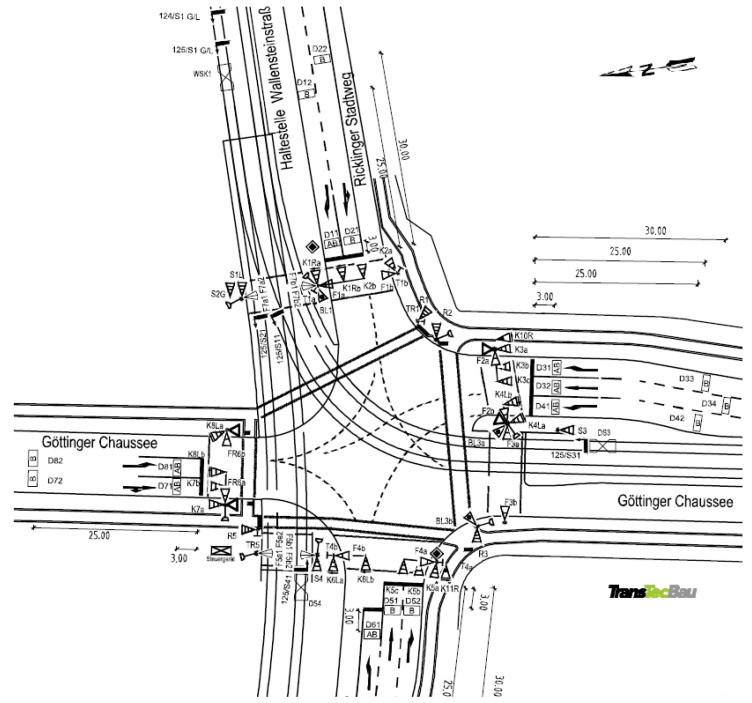
Signalized intersection LSA 125 in Hanover.

In order to train machine learning models, a large amount of traffic data needs to be collected by detectors implemented in the traffic system. As shown in Figure 2, the signalized Intersection 125, locating at the junction of Wallensteinstraße and Göttinger Chaussee in Hanover, is selected for research. It is a complex 4-leg intersection including footpaths, bike lanes, motorways, and tram tracks.

#### 2.1.1. Data Processing

The originally collected data is shown in Table 1, including all signal light states, requests from all traffic groups, detector data, timers, and so on. This table records the data every second for one whole day, including, in total, 86,400 s (rows) and 171 features (columns).

In Table 1, only traffic light states are labelled with characters, where “R” means the RED signal, “G” means the GREEN signal, “A” means the Attention (YELLOW) signal, “a” is the Acoustic signal for the blind, “S” means Start, “D” means the Dark signal. In order to train machine learning models, the data sheet has to be transformed into a full numerical table. Because only the releasing signals (i.e., “G” and “a”) mean the traffic participants are free to go through the intersection, they should be set to 1, while others have to be set to 0. If the detectors receive requests for crossing, it is set to 1. A full numerical data sheet after transformation is shown in Table 2.

Obviously, it is not necessary to take all features into consideration because the all-zero and redundant features not only seriously disrupt the forecasting accuracy but also heavily burden the calculation and training capability. After the filtration of features, the target to forecast in this work is selected as *K*01*R*, which is a signal controlled for one motor way in Figure 2, marked by *K*1*Ra* and *K*1*Rb*. After researching the phase logic installed in signal controllers, the key factors stimulating the changes among different phases are found. These key features are, respectively, the requests to cross (‘*ANF*’), detector data (‘*BK*’), approach timers (‘*TAN*’), emergency requests (‘*AFS*’), and reporting points (‘*MPN*’). After refining, the number of features is cut down to 38.

#### 2.1.2. Time Window Generation

**Figure 3:**
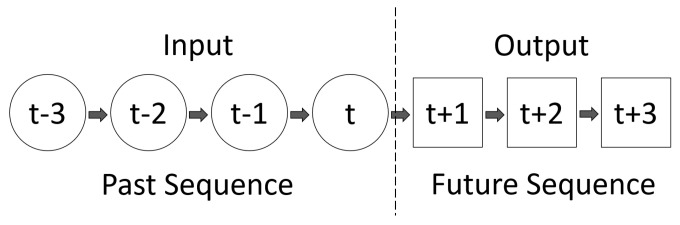
Multi-horizon direct forecasting method.

In this research, the signal prediction is viewed as a time-series problem. The multi-horizon direct forecasting method is adopted, which means a future sequence could be forecasted directly by an input of a historical observed sequence (see [14]). The sequence-to-sequence structure is described in Figure 3.

**Figure 4:**
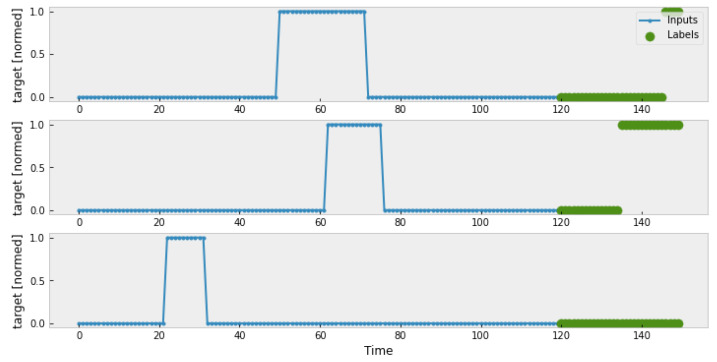
Generated time window.

The next step is to define the input sequence and forecasting sequence, which is named Time Window. As shown in Figure 4, the window size is set to be 120 s, the forecasting sequence is 30 s. The input historical observations are marked by blue points, the practical labels are marked by green points. And every time one round of prediction is finished, the time window slides forward for 30 s, until all timestamps are forecast.

In this work, 70% of data is selected to be the training set, 20% is the validation set, and the remaining 10% is selected for testing.

### 2.2. Machine Learning Models

Five basic machine learning models are trained for signal prediction, which are the Baseline Model, Linear Model, Dense Model, Convolutional Neural Network (CNN), and Long Short-Term Memory (LSTM). Of these, the Baseline Model and the Linear Model are selected as benchmark models for further comparison with others. All these models are built with Tensorflow on Google Colab. To train these models, some parameters are defined as:

- input_width = 120;
- OUT_STEPS = 30;
- num_features = 38;
- batchsize = 32;
- max_epoch = 32;
- patience = 5.

#### 2.2.1. Baseline Model

**Figure 5:**
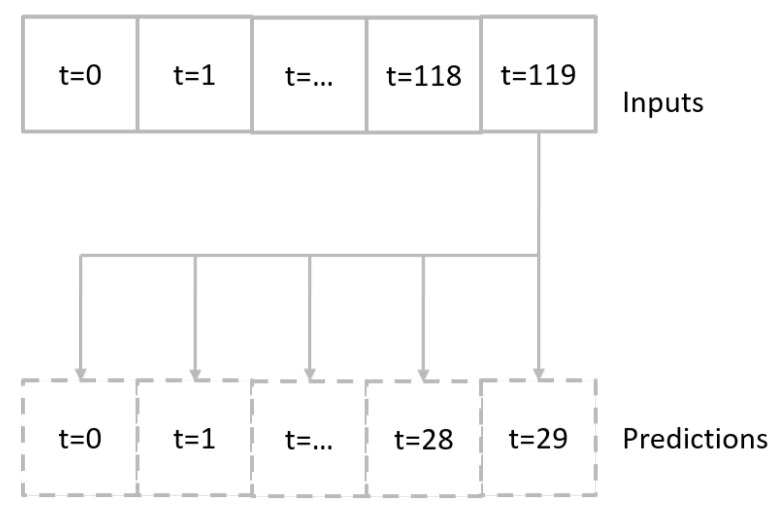
Construction of Baseline Model [15].

The baseline model adopted in this work is the Last Baseline Model. As shown in Figure 5, the predictions are only a repetition of the last seen input time step [15].

#### 2.2.2. Dense Model

**Figure 6:**
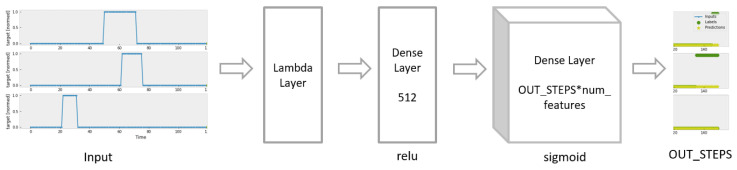
Construction of Dense Model.

The Dense Model is constructed as in Figure 6. It includes one fully connected layer with 512 output units, the relu activation function and one dense layer with the sigmoid activation function.

#### 2.2.3. Linear Model

**Figure 7:**
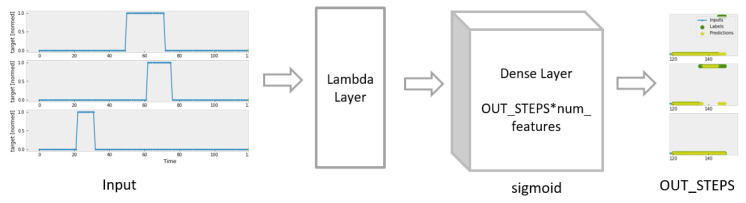
Construction of Linear Model.

Linear Model has only a simple linear layer which can be viewed as a simplified Dense Model (see Figure 7).

#### 2.2.4. CNN Model

**Figure 8:**
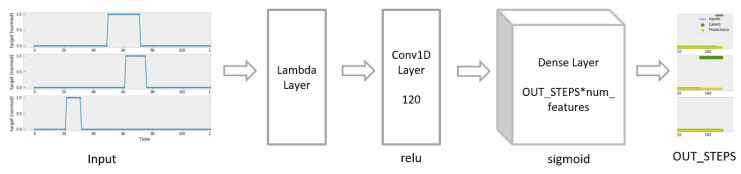
Construction of CNN Model.

As shown in Figure 8, the CNN Model consists of a one-dimensional convolution layer and one fully connected layer with the sigmoid activation function. The number of output filters in the convolution is 120.

#### 2.2.5. LSTM

**Figure 9:**
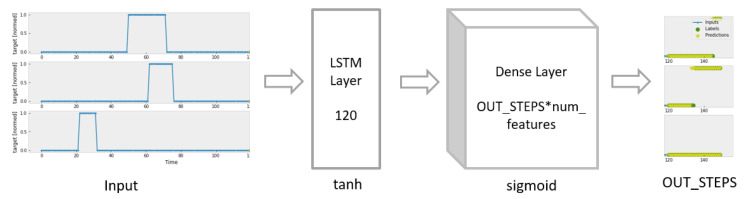
Construction of LSTM Model.

LSTM, a kind of Recurrent Neural Network, is viewed as one of the most promising approaches to forecast future time-series. As shown in Figure 9, it consists of an LSTM layer with 120 units and a fully connected layer.

**Figure 10:**
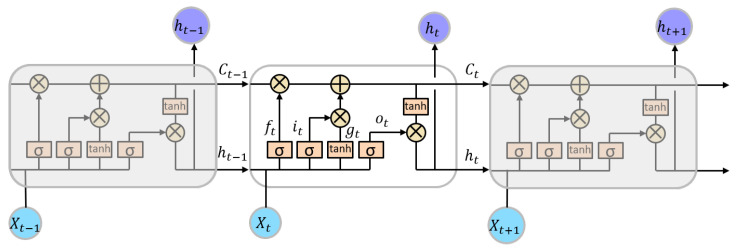
Internal structure of LSTM modules [16].

The internal structure of LSTM and the connections are presented in Figure 10, where $X_{t}$ means the input of feature matrix at current moment; similarly, $X_{t-1}$ and $X_{t+1}$ are inputs at the last and the next moment. The hidden layer outputs are represented by $h_{t-1}$, $h_{t}$, and $h_{t+1}$. $C_{t}$ is the internal memory state of the module which is called the cell state. Normally, LSTM modules are connected in a form of chains. It consists of a forget gate, an update gate, and an output gate.

The forget gate is represented by $f_{t}$ which decides how much information from the previous state should be forgotten. As described in Equation (1), $f_{t}$ is a number between 0 and 1, which can be calculated by $X_{t}$ and $h_{t-1}$, where *W* is a weight matrix and *b* is a bias.

(1) $$f_{t}=\sigma \left(W_{xf}X_{t}+W_{hf}h_{t-1}+b_{f}\right).$$

The update gate includes two parts, the input update $i_{t}$ and the candidate cell state $g_{t}$. $i_{t}$ decides how much new information should be updated (see Equation (2)). $g_{t}$ provides new candidate values that can be updated (see Equation (3)).

(2) $$i_{t}=\sigma \left(W_{xi}X_{t}+W_{hi}h_{t-1}+b_{i}\right),$$

(3) $$g_{t}=tanh\left(W_{xg}X_{t}+W_{hg}h_{t-1}+b_{g}\right).$$

A new cell state $C_{t}$ is generated after a combination of the forget gate and the update gate (see Equation (4)).

(4) $$C_{t}=C_{t-1}⊙f_{t}+g_{t}⊙i_{t}.$$

After the obtainment of the new cell state $C_{t}$, the output gate $o_{t}$ decides which part of the cell state $C_{t}$ should be output as a hidden layer output $h_{t}$ (see Equation (5)).

(5) $$\begin{array}{cc} o_{t}= & \sigma (W_{xo}X_{t}+W_{ho}h_{t-1}+b_{o}),\\ h_{t}= & o_{t}⊙tanh(C_{t}). \end{array}$$

Finally, the predicted future values of the time-series $y_{t}$ can be obtained by Equation (6).

(6) $$y_{t}=W_{yh}h_{t}+b_{y}.$$

## 3. Results

### 3.1. Comparison Results

#### 3.1.1. Binary Accuracy

**Figure 11:**
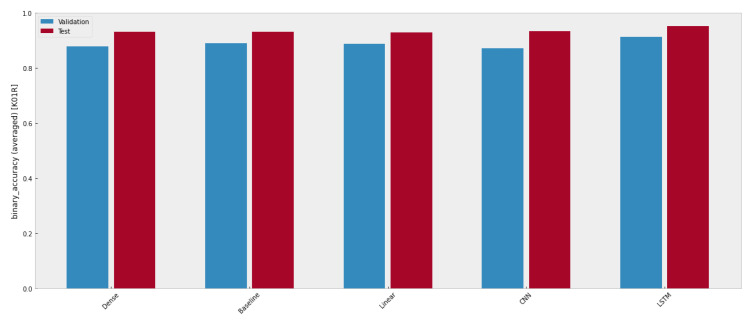
Comparison results of models trained with one-day data.

The binary accuracy of these five machine learning models is depicted in Figure 11, where the validation accuracy is marked by a blue bar, while the red bar represents the test accuracy. Though all models have similar forecasting ability, only the ones performing better than benchmark models should draw attention.

More intuitively, both test accuracy and validation accuracy are listed in Table 3. Obviously, LSTM is the only model that outperforms the others, which means LSTM is optimal to be selected for signal forecasting.

#### 3.1.2. Basic Metrics

As described above, for most of time, traffic signals are labelled with 0. In other words, there is a possibility that if the predictions are always set to be 0, a high binary accuracy can be obtained. In order to avoid this case, basic metrics are adopted to further evaluate these models. The related parameters are calculated in Equation (7), where TP is the number of True Positive predictions (i.e., the real values of 1 are predicted as 1), TN is the number of True Negative predictions (i.e., the real values of 0 are predicted as 0), FP is the number of False Positive predictions (i.e., the real values of 0 are predicted as 1), FN is the number of False Negative predictions (i.e., the real values of 1 are predicted as 0).

(7) $$\begin{array}{cc} ACC= & (TP+TN)/(TP+FP+FN+TN),\\ TPR= & TP/(TP+FN),\\ PPV= & TP/(TP+FP),\\ F1= & \frac{2}{\frac{1}{TPR}+\frac{1}{PPV}},\\ MCC= & \frac{TP\times TN-FP\times FN}{\sqrt{\left(TP+FP)(TP+FN)(TN+FP)(TN+FN\right)}}. \end{array}$$

As calculated by Equation (7), the accuracy ACC describes how much data are correctly predicted, but when the proportion of one and zero is unbalanced, this cannot reflect the real prediction situation. TPR is the True Positive Rate which describes how much actual one data is correctly predicted. For all data predicted as one, the PPV (Positive Predictive Value) describes the ratio of correct prediction. Because of the drawbacks of ACC, F1 score is calculated as a Harmonic Mean of TPR and PPV, which can better describe the prediction accuracy. MCC is Matthew’s correlation coefficient, which ranges between −1 and 1. If MCC equals zero, it usually means totally random predictions, while one means a perfect classifier. After calculation, the basic metrics of the researched machine learning models are listed in Table 4.

As shown in Table 4, even though both the Dense model and the CNN model have a high ACC of over 89%, their other metrics are 0. It means the prediction has no True Positive values. Obviously, LSTM outperforms other models for each metric.

**Figure 12:**
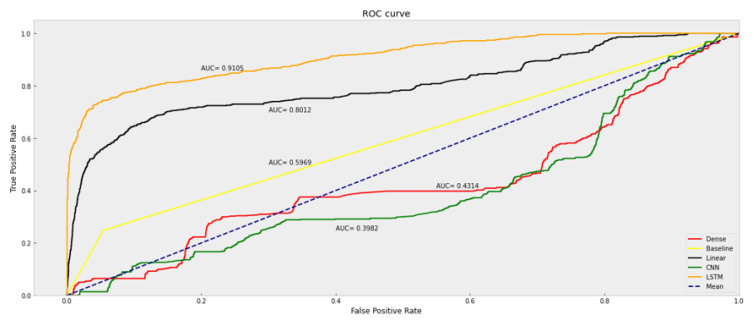
ROC curve of researched machine learning models.

In order to further analyze the diagnostic ability of a binary classifier system when the discrimination threshold varies, a receiver operating characteristic (ROC) curve is adopted. As shown in Figure 12, the ROC curve is created by plotting TPR against FPR as various threshold settings. The Area under the Curve (AUC) is further calculated to evaluate the classification ability. When the AUC score is 0.5, it means a totally random prediction, while a perfect classifier has one as the AUC score. It is more intuitive to find that LSTM performs the best compared with other models.

### 3.2. LSTM Trained by Data of One Month

**Figure 13:**
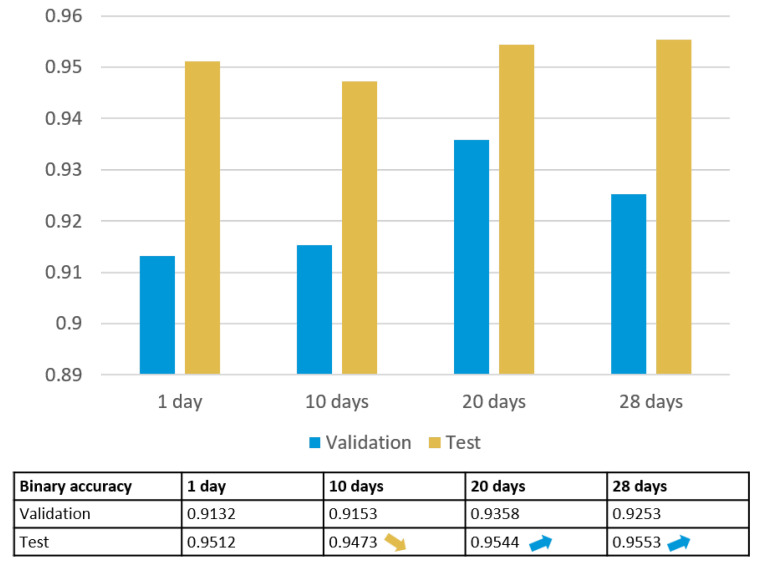
Training results of LSTM for different periods.

Because of the excellent forecasting performance of LSTM based on one-day data, a further training with one-month data of February follows. Rather than direct training with one-month data, the data sheet is divided into three groups to observe the accuracy changes in detail. After calculation, the training results of 1 day, 10 days, 20 days, and 28 days are depicted in Figure 13. It is interesting to find that the test accuracy reduces a little bit when the data horizon is extended from 1 day to 10 days. This could be caused when the model is trained well for one-day data (e.g., workday), but it may not predict well for another day (e.g., weekend). Nevertheless, as the data cover more time horizons, the corresponding general predicting accuracy increases. But the accuracy of one month is not improved significantly.

#### 3.2.1. Deviation Calculation

**Figure 14:**
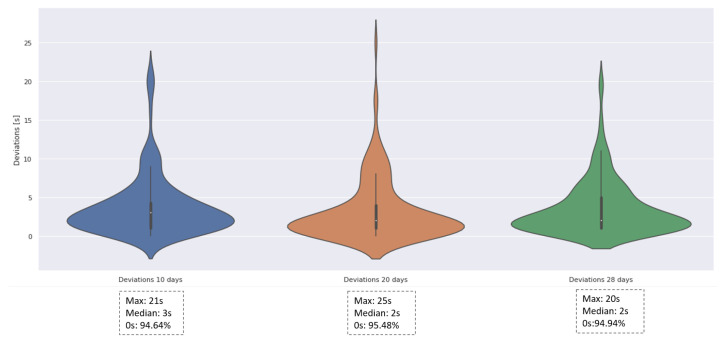
Violin plot of deviations for different training periods.

Since these machine learning models are applied for traffic signal prediction, one of the most vital evaluation indicators is the time deviation. Even an error of a few seconds can probably cause a serious traffic accident, especially at the moment of signal changes. Therefore, the deviations of the LSTM model trained by data of 10 days, 20 days, and 28 days are calculated for comparison. As shown in Figure 14, the violin plot can describe the distribution of deviations. The flatter the shape of the violin plot and the lower the median, the more concentrated and lower the deviations. The reason that the maximum deviation is approximate 20 s is that the model probably misses one GREEN signal, since it is really hard to guarantee the trained LSTM can catch each future signal changes with a 100% accuracy. However, the median of deviations is approximately 2 s, which means if the deviations exist, 50% of them are below 2 s. Even though the deviations cannot be avoided, about 95% of forecasting signals have no deviations. From a general perspective, there is a slight tendency that the longer horizon of the training data, the better the forecasting quality.

#### 3.2.2. Segment Accuracy Calculation

Another attractive evaluation indicator is the segment accuracy. In this work, the forecasting sequence of 30 s is cut into three equal segments to study whether the accuracy will be influenced by the length of forecasting horizon. These segments are defined as follows, where *t* represents the current day and t+1 represents the next day:

- Segment 1: t+1 -> t+10;
- Segment 2: t+11 -> t+20;
- Segment 3: t+21 -> t+30.

**Figure 15:**
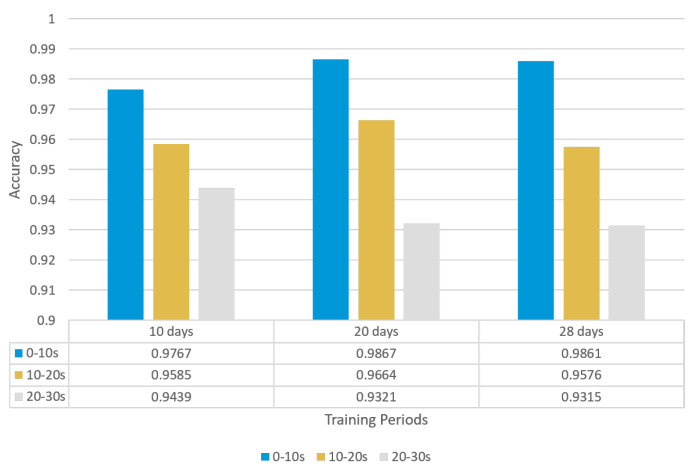
Segment accuracy for different data horizons.

The forecasting accuracies for these three segments are calculated, respectively, for three data horizons. As shown in Figure 15, due to the usage of their own test set, there is a vibration of forecasting accuracy for these three data horizons. However, it is obvious that the closer the segment to the current time *t*, the higher the accuracy, with the accuracy of these three segments showing a gradient downwards. As a result, such machine learning models cannot predict a really long future series.

#### 3.2.3. Basic Metrics of LSTM

**Figure 16:**
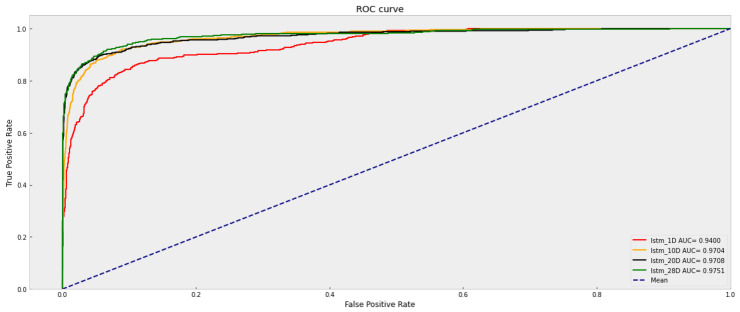
ROC curves of LSTM with different time horizons.

Similarly, related basic metrics of LSTM for different time horizons are calculated. As shown in Table 5, with the time horizons expanding, the accuracy increases. For a more intuitive view, the ROC curves of LSTM with different time horizons are depicted in Figure 16.

As shown in Figure 16, there is a significant improvement of AUC from the horizon of 1 day to 10 days. However, from 10 days to 28 days, the difference is not so obvious. That means that considering the training time and the complexity of the model, LSTM trained by 10-day data can be a good choice for further use.

## 4. Discussion

This paper provides adequate results that LSTM has a satisfactory performance in time-series forecasting problems with a test accuracy of over 95%. Further validation is performed to calculate the basic metrics of the researched models, including ACC, PPV, TPR, the F1 score, and MCC, all of which prove that the LSTM model outperforms other compared models for time-series forecasting. Furthermore, the ROC curves of LSTM for different horizons are drawn and show that LSTM trained by more than 10 days can have a significant improvement in terms of accuracy, while the differences among 10 days, 20 days, and 28 days are not so obvious. Another finding is that the deviations between the forecasting sequence and practical traffic signals should draw more attention, since there is still a quantity of deviations located at a high level. And the intersection chosen for research in this paper has no detectors for requests from buses. Therefore, the future work should focus on the development of a hybrid model of LSTM to narrow down the deviations to a reasonable range and find the influence of public transport when it is assigned priorities on the road, which will impact directly the traffic actuated signals. There is another situation that could not be neglected: When accident or jam happens, how will the prediction accuracy change? Theoretically, the vehicles can be detected by sensors, which will be input as a feature value. But due to lack of accident or jam data to train the machine learning models, the performance of these models cannot be verified in this work.

## Figures and Tables

**Table 1:** Collected data of one day.

Timestamp	K01R ^1^	K02	K03	K04L	K05	⋯	MPN_2	MPN_1
0	R	R	G	G	R	⋯	0	0
1	R	R	A	G	R	⋯	0	0
2	R	R	A	G	R	⋯	0	0
3	R	R	A	G	R	⋯	0	0
4	R	R	R	G	R	⋯	0	0
⋯	⋯	⋯	⋯	⋯	⋯	⋯	⋯	⋯
86,395	G	R	R	R	R	⋯	0	0
86,396	G	R	R	R	R	⋯	0	0
86,397	A	R	R	R	R	⋯	0	0
86,398	A	R	R	R	R	⋯	0	0
86,399	A	R	R	R	R	⋯	0	0

^1^ K01R is marked by K1Ra and K1Rb in Figure 2.

**Table 2:** Transformed full numerical data of one day.

Timestamp	K01R	K02	K03	K04L	K05	⋯	MPN_2	MPN_1
0	0	0	1	1	0	⋯	0	0
1	0	0	0	1	0	⋯	0	0
2	0	0	0	1	0	⋯	0	0
3	0	0	0	1	0	⋯	0	0
4	0	0	0	1	0	⋯	0	0
⋯	⋯	⋯	⋯	⋯	⋯	⋯	⋯	⋯
86,395	1	0	0	0	0	⋯	0	0
86,396	1	0	0	0	0	⋯	0	0
86,397	0	0	0	0	0	⋯	0	0
86,398	0	0	0	0	0	⋯	0	0
86,399	0	0	0	0	0	⋯	0	0

**Table 3:** Binary accuracy of machine learning models trained with one-day data.

Models	Validation Accuracy	Test Accuracy
Dense	0.8782	0.9325
Baseline	0.8904	0.9312
Linear	0.8869	0.9303
CNN	0.8722	0.9331
LSTM	0.9134	0.9525

**Table 4:** Basic metrics of researched machine learning models.

Models	ACC	PPV	TPR	F1	MCC
Dense	0.894	0.000	0.000	0.000	0.000
Baseline	0.856	0.277	0.227	0.249	0.172
Linear	0.911	0.722	0.249	0.371	0.390
CNN	0.894	0.000	0.000	0.000	0.000
LSTM	0.946	0.887	0.558	0.685	0.678

**Table 5:** Basic metrics of researched machine learning models.

Models	ACC	PPV	TPR	F1	MCC
LSTM_1D	0.924	0.912	0.449	0.602	0.609
LSTM_10D	0.949	0.922	0.656	0.766	0.752
LSTM_20D	0.962	0.957	0.736	0.832	0.820
LSTM_28D	0.962	0.966	0.726	0.829	0.819

## Data Availability

All researched data can be found at https://github.com/AmberXie/LOGIN (accessed on 4 July 2023).

## Abbreviations

The following abbreviations are used in this manuscript:

ACC	Accuracy
AUC	Area under the Curve
CNN	Convolutional Neural Network
FN	False Negative
FP	False Positive
FPR	False Positive Rate
IoT	Internet of Things
LSTM	Long Short-Term Memory
MAP	MAP as intersection geometry
MCC	Matthew’s Correlation Coefficient
PPV	Positive Predictive Value
ROC	Receiver Operating Characteristic
SPAT	Signal Phase and Timing
TN	True Negative
TP	True Positive
